# The effect of isohydric hemodialysis on the binding and removal of uremic retention solutes

**DOI:** 10.1371/journal.pone.0192770

**Published:** 2018-02-22

**Authors:** Aleksey Etinger, Kumar, William Ackley, Leland Soiefer, Jonathan Chun, Prabjhot Singh, Eric Grossman, Albert Matalon, Robert S. Holzman, Bjorn Meijers, Jerome Lowenstein

**Affiliations:** 1 NYU School of Medicine, NY, NY, United States of America; 2 Dept. Nephrology, University Hospitals Leuven, Leuven, Belgium; 3 Dept. Immunology and Microbiology, Leuven, Belgium; University Medical Center Utrecht, NETHERLANDS

## Abstract

**Background:**

There is growing evidence that the accumulation of protein- bound uremic retention solutes, such as indoxyl sulfate, p-cresyl sulfate and kynurenic acid, play a role in the accelerated cardiovascular disease seen in patients undergoing chronic hemodialysis. Protein-binding, presumably to albumin, renders these solutes poor-dialyzable.

We previously observed that the free fraction of indoxyl sulfate was markedly reduced at the end of hemodialysis. We hypothesized that solute binding might be pH-dependent and attributed the changes in free solute concentration to the higher serum pH observed at the end of standard hemodialysis with dialysis buffer bicarbonate concentration greater than 35 mmol/L. We observed that acidification of uremic plasma to pH 6 in vitro greatly increased the proportion of freeIS.

**Methods:**

We tested our hypothesis by reducing the dialysate bicarbonate buffer concentration to 25 mmol/L for the initial half of the hemodialysis treatment (“isohydric dialysis”). Eight stable hemodialysis patients underwent “isohydric dialysis” for 90 minutes and then were switched to standard buffer (bicarbonate = 37mmol/L). A second dialysis, 2 days later, employed standard buffer throughout.

**Results:**

We found a clearcut separation of blood pH and bicarbonate concentrations after 90 minutes of “isohydric dialysis” (pH = 7.37, bicarbonate = 22.4 mmol/L) and standard dialysis (pH = 7.49, bicarbonate = 29.0 mmol/L). Binding affinity varied widely among the 10 uremic retention solutes analyzed. Kynurenic acid (0.05 free), p-cresyl sulfate (0.12 free) and indoxyl sulfate (0.13 free) demonstrated the greatest degree of binding. Three solutes (indoxyl glucuronide, p-cresyl glucuronide, and phenyl glucuronide) were virtually unbound. Analysis of free and bound concentrations of uremic retention solutes confirmed our prediction that binding of solute is affected by pH. However, in a mixed models analysis, we found that the reduction in total uremic solute concentration during dialysis accounted for a greater proportion of the variation in free concentration, presumably an effect of saturation binding to albumin, than did the relatively small change in pH produced by isohydric dialysis. The effect of pH on binding appeared to be restricted to those solutes most highly protein-bound.

The solutes most tightly bound exhibited the lowest dialyzer clearances. An increase in dialyzer clearance during isohydric and standard dialyses was statistically significant only for kynurenic acid.

**Conclusion:**

These findings provide evidence that the binding of uremic retention solutes is influenced by pH. The effect of reducing buffer bicarbonate concentration (“isohydric dialysis:”), though significant, was small but may be taken to suggest that further modification of dialysis technique that would expose blood to a greater decrease in pH would lead to a greater increase the free fraction of solute and enhance the efficacy of hemodialysis in the removal of highly protein-bound uremic retention solutes.

## Introduction

Hemodialysis was established as an effective technique for maintaining patients with end-stage renal disease by Belding Scribner and his associates [1], but systematic investigation of the nature of uremic toxins is usually dated to the report of the European Uremic Toxin Workshop (EUTox) group which surveyed a wide range of published studies and defined uremic toxins as substances whose concentration in blood was significantly higher in patients with evidence of reduced glomerular filtration [2]. Over the ensuing years, techniques, generally employing high pressure liquid chromatography (HPLC), coupled with mass spectroscopy (MS), have identified a great number of solutes that are at higher plasma concentration in patients with evidence of decreased glomerular filtration (estimated by a variety of techniques). These direct measurements have generally confirmed the conclusion of the original EUTox report that these solutes fall into three classes (1) small dialyzable water soluble molecules such as urea or creatinine, (2) larger molecules, from a historical perspective often referred to as “middle molecules”, such as beta-2 microglobulin and complement, defined as being too large to pass across conventional dialysis membranes, and (3) small protein-bound solutes that do not readily diffuse across conventional dialysis membranes. The number and identity of these uremic solutes differ somewhat depending on the platform employed to identify them. As the identification of a toxin requires direct evidence of toxic effects, the term “uremic retention solutes” (URS), is preferable and has largely replaced the older term “uremic toxins”. Among the many known uremic retention solutes, the more highly protein-bound indoxyl sulfate (IS), p-cresyl sulfate (PCS), and kynurenic acid (KA) appear to be true uremic toxins. Observational studies have reported an association between the plasma concentration of these uremic retention solutes and the incidence of what has been termed “uremic accelerated cardiovascular disease”, characterized by heart failure, cardiac arrhythmias, and sudden death [3–7]. A recent analysis of the relationship between the concentration of indoxyl sulfate and p-cresol sulfate and cardiovascular events in plasma samples collected during the HEMO study failed to confirm the association in patients with serum albumin concentration greater than 34 g/L[8]. A commentary in the same issue suggested that “analytical issues and case-mix might explain the discrepant findings [9].

Given that these solutes are poorly dialyzable, attention has focused on alternate modes of reducing their plasma concentration [10,11,12,13]. In a prior study we observed that the concentration of free (free) IS was markedly decreased at the conclusion of standard hemodialysis [14]. The dependence of solute binding to protein (albumin) on pH is a well- established phenomenon [15]. In an in vitro pilot study, we observed that acidification of 3 dialysis plasma samples, by addition of acetate buffer with pH = 6, increased free indoxyl sulfate concentrations from values of 8 μmol/L to 61 μmol/L, 9.9 μmol/L to 117 μmol/L and from below the limit of detection to 44.0 μmol/L [14]. We felt that this in vitro study together with existing evidence that binding to albumin of many solutes is influenced by pH [15] prompted us to proceed to study patients undergoing hemodialysis, a procedure we knew to be associated with an increase in serum bicarbonate concentration and pH. We confirmed that during hemodialysis utilizing a standard buffer with bicarbonate concentration greater than plasma led to increased pH and plasma bicarbonate. Arterial pH and bicarbonate concentration increased during hemodialysis, approaching a new steady state midway through a standard hemodialysis treatment [14]. This led us to examine the effects of preventing the increase in blood pH that occurs during standard hemodialysis by reducing the dialysis buffer bicarbonate concentration (“isohydric hemodialysis”).

## Material and methods

### Patients

Eight stable patients with end-stage renal failure were recruited in the River Renal Dialysis Unit in Bellevue Hospital, New York City (Table 1). They were undergoing thrice weekly dialysis sessions via an arteriovenous fistula. Exclusion criteria were age less than 18 years, pregnancy, inability to provide informed consent, incarceration, hospitalization within the past 12 months, serum potassium < 4.0 mmol/L or corrected serum calcium < 4.25 mmol/L on the previous monthly test. The study was approved by the New York University School of Medicine and Bellevue Hospital Institutional Review Boards. All participants provided witnessed, written informed consent to participate in this study.

**Table 1:** Clinical characteristics of subjects.

Subject	Age (yr)	Gender	Ethnicity	Months of Hemodialysis	Etiology of Renal Disease	Heparin with Hemodialysis
A	40	F	African American	25	SLE	No
B	71	M	Asian	26	IgA	Yes
D	59	M	African American	56	HIVAN	Yes
E	58	M	African American	30	HTN	Yes
F	66	M	Caucasian	65	HTN	Yes
G	46	M	African American	77	HTN	Yes
H	41	M	Hispanic	33	DM	No
I	67	M	African American	37	HTN	Yes

SLE = Lupus nephritis, IgA = IgA nephropathy, HIVAN = HIV nephropathy, HTN = hypertensive nephropathy, DM = diabetic nephropathy

### Study design

Each patient underwent a mid-week “isohydric dialysis” followed by a “standard dialysis” two days later. Isohydric dialysis was performed with buffer bicarbonate concentration of 25 mmol/L for the initial 90 minutes, followed by dialysis with standard buffer (bicarbonate 37 mmol/L) for 90 minutes. Standard dialysis utilized buffer (37 mmol/L) throughout. Subject G was withdrawn from the study after the “isohydric dialysis”, when he failed to attend the standard dialysis session. He had no adverse reaction to the isohydric dialysis 2 days earlier. Dialyses were performed utilizing single use F160NR or F180NR, polysulfone-based membranes (Fresenius Medical Care, Waltham, Massachusetts), depending on each patient’s pre-arranged clinical management plan. The “standard”dialysate contained sodium 138 mmol/L, chloride 108.5 mmol/L, calcium 2.5 mmol/L, bicarbonate 37 mmol/L, and acetate 3.0 mmol/L. The composition of the “Isohydric” dialysate was identical except that bicarbonate was reduced to 25 mmol/L. Chloride ions replaced the removed bicarbonate. Dialysate flow rate was 800 mL/min. Dialyzer blood flow ranged from 300 to 500 mL/min. Unfractionated heparin was used as needed in all but two of the patients. These parameters for dialysate and blood flow have been standard in the River Renal Dialysis unit for many years.

Blood samples (5 ml) were collected from the line entering the dialyzer (arterial blood) at the beginning of each dialysis session and every 45 minutes thereafter. Arterial blood gas was measured and bicarbonate calculated using a Siemens ABL90 blood gas analyzer (40 Liberty Boulevard Malvern, PA USA). Blood samples for measurement of uremic retention solutes were collected in EDTA anticoagulant and placed on ice until centrifugation at 1500 rpm for 10 minutes. The plasma supernatant was stored in cryovials at -80°C. Ultrafiltration was performed utilizing a Millipore 3kDa Centrifugal Filter (Sigma-Aldrich St. Louis, MO USA) at 14,000 rpm for 30 minutes. Uremic retention solutes in plasma and plasma ultrafiltrate were measured by the MS- HPLC method of De Loor [16]. Values for the concentration of retention solutes in ultrafiltrates did not differ between subjects who did and did not receive heparin during hemodialysis.

During both Isohydric and standard dialyses, a sample of spent dialysate was collected at the 90-minute midpoint. Five ml aliquots of dialysate were stored in cryovials at -80°C.

The degree of protein binding was assessed by computing the fraction of solute that was free according to the following equation.

$$Free\;Fraction=\frac{Free\;Concentration(\frac{\mu M}{L})}{Total\;Concentration\;(\frac{\mu M}{L})}$$ (1)

The dialyzer clearance for each uremic solute was calculated as;

$$Dialyzer\;Clearance=U_{Solute}\times V/P_{Solute}$$ (2)

where U represents the concentration of solute in the dialysate, V represents the dialysis flow rate (fixed at 800 mL/min) and P represents the mean of the total solute concentrations at 45 and 90 minutes. This calculation estimates the dialyzer clearance of solute in the same manner as UV/P for creatinine estimates the renal clearance of creatinine in those not receiving dialysis.

### Statistical analysis

Analytic data were entered into an Excel spreadsheet and imported into the R statistical environment [17]. To analyze the changes over time within subjects, and allow for any missing data, mixed models analyses were conducted using R’s lme4 package [18]. We considered time, pH, solute concentrations, and free fraction to be fixed factors (i.e. factors specified or determined by the experimental design) and subjects as a random factor (i.e. a sample taken from some larger group of possible subjects and therefore subject to sampling variation and a source of within-subject correlations in repeated measurements of the fixed variables). Separate analyses were conducted for each solute to estimate average plasma concentrations, dialysate concentrations, free fractions, and clearances.

Differences of interest were examined by analysis of variance. Following a significant overall analysis, adjusted means (i.e. group means as computed from the model equation) were computed and tested for significance using R’s lsmeans package [19]. Tukey’s “Honestly Significant Difference” adjustment was used for all *post hoc* tests to hold the family-wise type I error rate at 5%. The proportion of variation accounted for by specific factors in various mixed models was estimated using the method of Nakagawa and Scheilzeth [20].

All original data are available in supporting S1 File.

## Results

### pH and bicarbonate changes

**Fig 1:**
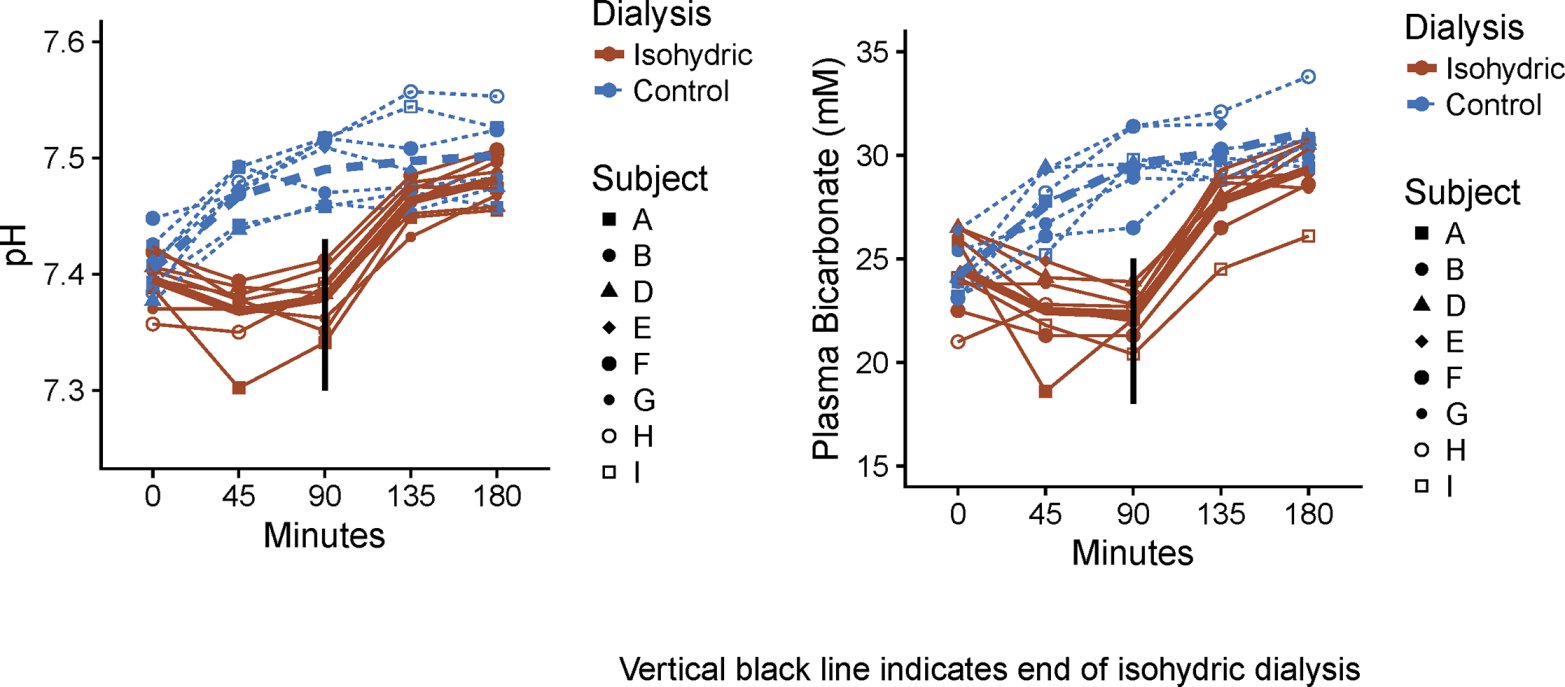
Changes in pH and bicarbonate concentration during standard and isohydric dialysis. Individual lines are actual subject data, heavy lines are adjusted means from mixed model. The black vertical line represents the time during isohydric dialysis at which the bicarbonate buffer concentration was changed.

The reduction in buffer bicarbonate concentration from standard (37 mmol/L) to “isohydric” (25 mmol/L) resulted in a clear and consistent difference in blood pH and bicarbonate concentrations. Table 2 summarizes the variation between subjects and Fig 1 the changes within each patient.

**Table 2:** Changes in pH and bicarbonate during isohydric and standard dialysis.

	Dialysis	0 Min	90 Min	Change after 90 Min
pH^1^	Isohydric	7.40 ± 0.02	7.38 ± 0.03	-0.02
	Standard	7.41 ± 0.02	7.49 ± 0.03	0.08
Bicarbonate (mmol/L) ^1^	Isohydric	24.5 ± 2.03	22.3 ± 1.13	-2.20
	Standard	23.8 ± 1.28	29.9 ± 1.67	6.1

^1^ Mean ± Standard Deviation

After 90 minutes of isohydric dialysis mean arterial pH declined slightly from 7.40 to 7.38 (difference = 0.02, standard error (SE) difference = 0.01, paired t (6 degrees of freedom (df)) = 1.58, p = NS), while during standard dialysis it increased significantly from 7.41 to 7.49 (difference = 0.08, SE difference = 0.01, paired t (5df) = 6.45, p < 0.001). During the same interval of isohydric dialysis mean plasma bicarbonate declined from 24.5 to 22.3 mmol/L (difference = 2.2, SE difference = 0.69, paired t (7df) = 3.21, p = 0.02) and during standard dialysis it increased from 23.8 to 29.9 mmol/L (difference = 6.1, SE difference = 0.79, paired t (5df) = 7.68, p<0.001). By mixed models analysis the difference between the change in pH seen after 90 minutes of isohydric dialysis, -0.08, and the change in pH seen after 90 minutes of standard dialysis, -0.02, was statistically significant (difference = 0.10, p < 0.001). In our previous study (14) we observed that pH increased from 7.39 to 7.52 and bicarbonate concentration increased from 24.2 to 31.6 mmol/L after 90 minutes of standard hemodialysis.

By design, the “standard dialysis” was always performed 2 days following the “isohydric dialysis”. The near identity of baseline values for pH and bicarbonate seen in isohydric dialysis and the standard dialysis that followed 2 days later is evidence that isohydric dialysis did not result in a measurable decrease in blood buffering.

### Protein binding, plasma concentrations, and clearance of uremic solutes

Table 3 lists for the 10 solutes, the changes in free fraction, mean total concentrations and plasma clearance [during isohydric and standard dialysis. As before, the table summarizes variation between patients while figures show changes by individual patient.

**Table 3:** Changes in free fraction. Solute concentration and plasma clearance during isohydric and standard dialysis.

Solute	Dialysis	Free Fraction		Total Solute Concentration^2^ (μmol/L)		Plasma Clearance (mL/Min)
		0 Min	90 Min	0 Min	90 Min	
Kynurenic acid (KA)	Isohydric	0.06 ± 0.02^1^	0.03 ± 0.01	1.60 ± 1.07	1.18 ± 1.08	58.14 ± 28.83^3^
	Standard	0.05 ± 0.01	0.02 ± 0.01	1.43 ± 0.72	1.08 ± 0.51	41.30 ± 28.99
p-Cresyl sulphate (PCS)	Isohydric	0.12 ± 0.06	0.08 ± 0.04	250.98 ± 66.16	217.83 ± 60.34	18.33 ± 6.09
	Standard	0.10 ± 0.07	0.09 ± 0.05	258.18 ± 84.71	214.14 ± 54.66	17.49 ± 4.88
Indoxyl sulphate (IS)	Isohydric	0.15 ± 0.06	0.10 ± 0.04	142.18 ± 52.08	116.56 ± 44.89	25.52 ± 7.75
	Standard	0.13 ± 0.07	0.11 ± 0.05	139.42 ± 55.45	113.04 ± 48.84	25.80 ± 7.11
Phenyl sulphate (PS)	Isohydric	0.38 ± 0.15	0.26 ± 0.11	35.96 ± 16.54	22.82 ± 10.25	48.95 ± 13.75
	Standard	0.35 ± 0.14	0.31 ± 0.10	35.67 ± 24.04	22.57 ± 17.22	48.56 ± 10.27
Kynurenine (KY)	Isohydric	0.41 ± 0.17	0.34 ± 0.17	4.26 ± 1.22	2.72 ± 0.77	108.50 ± 18.33
	Standard	0.33 ± 0.21	0.28 ±0.16	4.03 ± 1.30	2.63 ± 0.63	95.86 ± 26.52
Indole-3 acetic acid (IA)	Isohydric	0.51 ± 0.16	0.34 ± 0.12	7.94 ± 5.23	5.62 ± 3.62	68.77 ± 93.70
	Standard	0.47 ± 0.20	0.39 ± 0.11	7.76 ± 6.37	5.17 ± 4.00	69.86 ± 28.74
Hippuric acid (HA)	Isohydric	0.62 ± 0.09	0.48 ± 0.05	280.65 ± 168.45	121.85 ± 80.43	93.70 ± 53.55
	Standard	0.59 ± 0.07	0.46 ± 0.06	212.42 ± 154.92	96.67 ± 81.40	108.05 ± 18.33
Indoxyl glucuronide (IG)	Isohydric	0.83 ± 0.14	0.75 ± 0.11	5.51 ± 5.23	2.09 ± 3.62	130.25 ± 59.53
	Standard	0.85 ± 0.16	0.85 ± 0.12	5.55 ± 5.56	2.26 ± 2.39	168.80 ± 83.98
p-Cresyl glucuronide (PCG)	Isohydric	0.90 ± 0.04	0. 85 ± 0.06	20.83 ± 17.77	5.28 ± 3.26	149.04 ± 56.93
	Standard	0.87 ± 0.04	0.87 ± 0.03	21.86 ± 17.74	7.54 ± 6.34	130.85 ± 26.92
Phenyl glucuronide (PG)	Isohydric	1.00 ± 0.01	0.99 ± 0.02	0.95 ± 1.06	0.30 ± 0.31	108.49 ± 67.63
	Standard	0.99 ± 0.03	1.00 ± 0.00	1.16 ± 1.25	0.50 ± 0.62	138.09 ± 49.36

^1^Mean ± Standard Deviation

^2^All declines in concentration between 0 and 90 minutes were statistically significant in mixed models analyses (p < 0.001)

^3^Difference in clearance during isohydric and standard dialyses significant in mixed models analysis (p< 0.05).

#### Plasma concentrations

As shown in Table 3, plasma levels of all 10 solutes declined after 90 minutes of dialysis. The decline for each solute was statistically significant (p < 0.001) in mixed model’s analysis. The magnitude of the decline during isohydric dialysis was not statistically different than the decline during standard dialysis for any solute.

**Fig 2:**
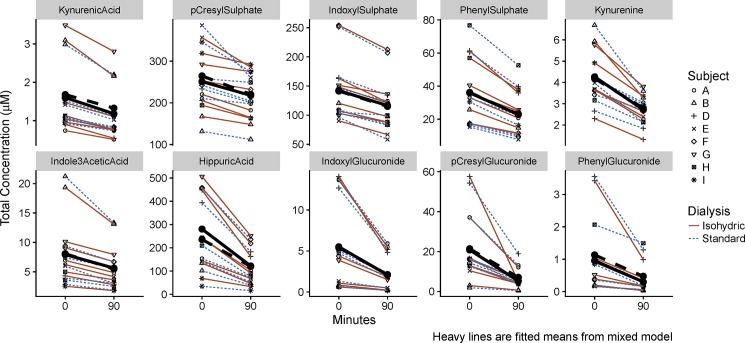
Changes in total solute concentration during isohydric and standard dialysis. Heavy lines represent fitted means from mixed model. Colored lines represent individual patient data.

Fig 2 shows, graphically, the changes in total solute concentration during isohydric and standard dialyses. Solutes are arrayed in order of decreasing protein binding. Among the ten solutes, the magnitude of reduction in concentration during both isohydric and standard dialysis appeared to be related to the degree of protein binding. Those solutes most highly protein-bound (KA, PCS, IS) had the smallest percent reduction in total solute concentration.

#### Protein binding

**Fig 3:**
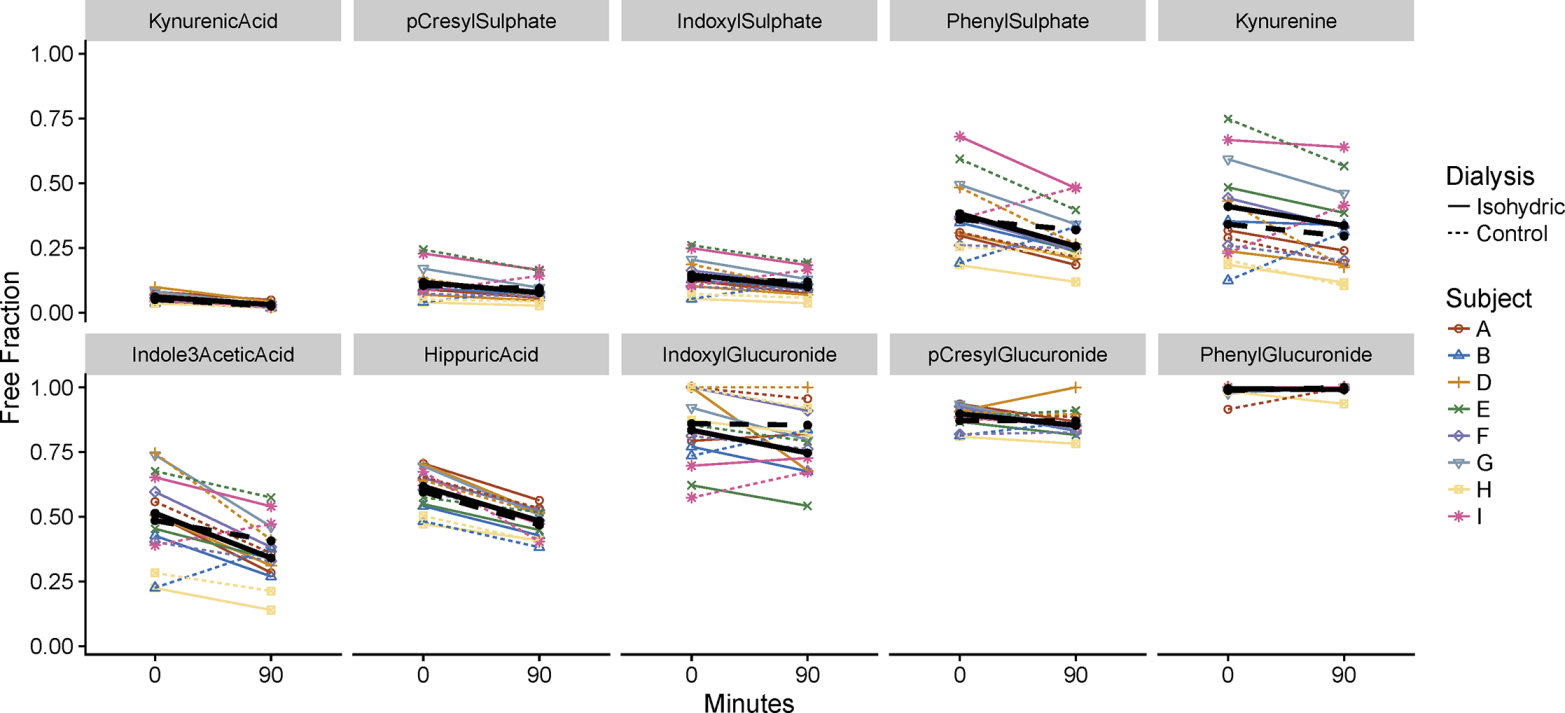
Changes in free fraction during isohydric and standard dialyses. Heavy lines connect fitted means from the mixed model analyses. Colored lines represent individual patient data.

Binding affinity, as assessed by the fraction of solute that was free, varied widely among the 10 uremic retention solutes analyzed. KA, PCS and IS demonstrated the greatest degree of binding. Three solutes (IG, PCG, and PG) were virtually free. Free fraction of the solutes generally decreased during the initial 90 minutes of dialysis, reflecting an increase in protein binding. Data are shown in Table 3 and Fig 3. Decreases were statistically significant, with p-values from the mixed models analyses between 0.49 and 7x10^-8^ for all solutes except IG (p = 0.138) and PG (p = 0.697). Decreases during isohydric dialysis did not differ significantly from changes during standard dialysis for any solute. The very low concentrations of free PCS and IS after dialysis (both isohydric and standard) confirm our earlier observations [14].

#### Clearance

The clearance of uremic solutes via the dialyzer was calculated as noted in the Methods.

Table 3 shows the mean clearances at 90 minutes of isohydric or standard dialysis. By mixed model’s analysis the difference between isohydric and standard dialyses was statistically significant only for Kynurenic acid (p< 0.05 by Tukey’s HSD). Notably, those solutes most tightly bound (KA, IS, and PCS) exhibited the lowest clearances. The observation that the clearance of kynurenic acid, the most highly bound of the uremic solutes studied, is greater than that of IS and PCS, the other highly-bound solutes, suggests that factors other than the extent of protein binding may affect dialyzer clearance of free solute.

### Relative contributions of pH and concentration to protein binding

We assessed the relative contributions of pH and total concentration of solute to determining the free solute concentration by creating mixed models and examining the proportion of variation (R squared) accounted for by pH or total solute concentration. For each solute, total plasma concentration was the dominant factor, accounting for an average of 76 percent (range 39 to 99) of the observed variation in free concentration while pH only accounted for an average of 10 percent (range 1 to 16). The data are summarized in Table 4.

**Table 4:** Proportion of variation accounted for by mixed models predicting free concentration from either pH or total concentration.

Solute	Proportion of Variation accounted for by	
	pH	Total Concentration
Kynurenic Acid	0.14	0.69
p-Cresyl sulphate	0.05	0.54
Indoxyl sulphate	0.10	0.60
Phenyl sulphate	0.13	0.69
Kynurenine	0.12	0.39
Indole-3-acetic acid	0.09	0.75
Hippuric acid	0.16	0.97
Indoxyl glucuronide	0.07	0.96
p-Cresyl glucuronide	0.11	0.99
Phenyl glucuronide	0.01	0.99
Mean ± Standard Deviation	0.10 ± 0.04	0.76 ± 0.21

## Discussion

We compared the effects of maintaining the baseline pH and bicarbonate of plasma during dialysis (“isohydric dialysis”) with those during standard dialysis, on protein binding and dialyzer removal of 10 solutes classified as uremic retention solutes. The binding, presumed to be at Sudlow site II, varied over a considerable range, from 0.95 bound for KA to near zero bound for PG (Table 3). Of note, the three solutes most highly bound, KA, IS, and PCS, exhibited free fractions that ranged from 0.055–0.15 despite very large differences in concentration of total solute (from .06μmol/L to 20μmol/L). Meijers et al [21] similarly reported that the free fractions of IS and PCS were very closely correlated over a considerable range of concentration of each solute and observed that spiking uremic plasma with either IS or PCS resulted in an increase in the free fraction of both solutes, suggesting a common binding site. Similar evidence of a shared binding site for PCS and IS was reported by Watanabe et al [22]. The solutes we studied appear to form 3 groups, highly bound (KA, PCS, IS), moderately bound (PS, KY, IAA) and poorly bound (HA, IG, PCG, PG). The observation that the uremic retention solutes that are considered uremic toxins (KA, IS, and PCS) exhibited the greatest affinity for albumin suggests that affinity for albumin and vascular receptors rather than free solute concentration may be the major determinant of toxicity. The reported Km for the binding of kynurenic acid to OAT1 is close to the Km for binding of several protein bound solutes to albumin [22, 23, 24].

There was an observable relationship between binding and reduction in concentration by dialysis. As shown in Table 2, total concentration of all the uremic retention solutes declined during both isohydric and standard dialysis. The decline was least in the 3 solutes with highest protein binding and greatest for the 3 with the lowest binding. The remaining four solutes exhibited intermediate levels of protein binding and removal. Overall these findings are consistent with the earlier reports by Rhee et al [25, 26]. Studying the dialytic removal of a panel of uremic retention solutes during a single dialysis, these investigators identified only KA and IS as “poorly dialyzed” when compared with virtually all the other measured uremic retention solutes which were removed as effectively as creatinine, i.e., as small readily dialyzed solutes. PCS was not identified in their MS-HPLC platform.

We observed that isohydric dialysis achieved by reduction of dialysis buffer bicarbonate concentration to 25 mmol/L prevented the observed increase in plasma pH and bicarbonate observed with standard hemodialysis. The findings described here confirm our expectation that the binding of uremic retention solutes to protein, most likely Sudlow site II on albumin [27], is pH-dependent. These isohydric dialysis studies demonstrate an effect of pH on solute binding, but the size of the effect, constrained as it was by the study’s intent to maintain an isohydric pH in the subjects by reducing buffer bicarbonate concentration, was considerably less than the magnitude of the effect attributable to the reduction in total solute concentration achieved by hemodialysis. These non-covalently bound solutes exhibit saturable binding to albumin and, predictably, when total solute concentration decreases during dialysis, the concentration of free solute decreases.

It is notable that we found that maintaining isohydric pH to affect the dialyzer clearance of KA, the solute with the greatest protein binding. Dialyzer clearance was significantly greater during isohydric dialysis than standard dialysis (Table 3). Among the remaining solutes examined in this study, binding affinity was lower and the effect of pH less evident or absent. This may be the most sensitive evidence of the effect of pH on binding of KA to albumin.

Prior studies have examined the effect of altering the ionic strength of plasma on the binding and dialysance of uremic solutes. Krieter et al [28] demonstrated that hemodiafiltration with increased plasma ionic strength (sodium concentration 240 mmol/L) resulted in 40% greater reduction (48.7 ± 23.6 vs. 67.8 ± 7.9%; P = 0.013) in free IS. Sodium concentration in arterial blood increased from 132 ± 2 to 136 ± 3 mmol/L. While the concentration of sodium in arterial blood was small, it would seem likely that total exchangeable sodium would be considerably increased. The authors noted “HDF-IPIS (hemodiafiltration at increased plasma ionic strength) is technically and clinically feasible. More effective HDF-IPIS requires higher temporary plasma [Na+], but dialysate [Na+] has to be appropriately adapted to avoid sodium accumulation”. As a proof of principle this study provides evidence that binding of indoxyl sulfate to protein (presumably albumin) can be altered by changing the ionic concentration of plasma but does not yet present a promising therapeutic approach. Bohringer et al [29] studied the in vitro effects of hypertonic predilution hemodiafiltration and observed greater free concentration of several uremic solutes when dialysate sodium concentration was increased from 140 mmol/L to1000 mmol/L. Again, while the effect of ionic strength clearly affected solute binding the technique does not appear to be readily applicable to treatment of dialysis-dependent patients.

The major drawback of our proposed modification of hemodialysis treatment was the small (although statistically significant) reduction in pH achieved by preventing the increase in pH that occurs during standard dialysis. The difference in pH after 90 minutes of isohydric dialysis as compared with standard dialysis, though significant, was small, averaging 0.11 pH units. The effects of isohydric dialysis are attributable to reduced bicarbonate concentration in the systemic circulation. Employing a reduced bicarbonate concentration in the dialysis fluid, we maintained a lower pH and bicarbonate in circulating blood and therefore in the blood entering the dialyzer. Whether the effect we have demonstrated can be exploited by further reducing the pH of blood passing through the hollow fiber kidney and thereby increasing free solute concentration and dialyzer removal of uremic solute deserves serious consideration. Two possibilities have been considered. The first would be to dialyze against a lower dialysate bicarbonate concentration. This would lower pH in the dialyzer by reducing bicarbonate concentration in the systemic circulation. Significant reduction in serum bicarbonate might cause adverse symptoms. Back titration to correct the induced metabolic acidosis following dialysis, would require close monitoring as bicarbonate loss might vary from subject to subject. A second approach might be to selectively acidify the blood during its passage through the dialyzer. When dialysis solutions containing acetic acid or citric acid are mixed with bicarbonate immediately before entering the dialyzer, CO_2_ is generated [30]. Sombolos et al [31] observed a PCO_2_ of 60.6–66.9 mm Hg in blood exiting the dialyzer during high flux dialysis. The PCO_2_ in systemic blood entering the dialyzer was consistently around 39.0 mm Hg, evidence that the CO_2_ added by the dialyzer was removed from the systemic circulation by the lungs. We are considering a method by which CO_2_ might be added to the blood entering the dialyzer by inserting an extracorporeal membrane oxygenator (ECMO) unit proximal to the dialysis cartridge. We think it is likely the alveolar ventilation would remove the added CO_2_ and obviate a change in systemic acid-base balance.

We acknowledge that there is no identified, clinically meaningful target concentration for any of these solute/toxins. However, the reported better outcomes (survival and quality of life) of patients with residual renal function (RRF) as compared with those who are anuric, suggest that a modest reduction in concentration may have clinical effects. Contrary to the view that RRF represents reabsorbed glomerular filtrate [32], many lines of evidence suggest that RRF represents, in whole or part, solute and fluid secreted by proximal renal tubular cells [33,34]. In hemodialysis [35] and in peritoneal dialysis [36] RRF was associated with better survival independent of the intensity of dialysis. Several studies have compared the concentrations of one or several uremic retention solutes in patients with RRF as compared with patients who are anuric [37, 38]. In our small published study we reported that indoxyl sulfate concentration averaged 56 μmol/L in anuric patients and 37 μmol/L in subjects with RRF [37], a difference of about 33% which may be attributed to the secretion of uremic solutes. This difference in concentration provides one estimate of a difference that may account for the observed differences in clinical, largely cardiovascular, outcomes. In light of the decrease in the plasma concentration of the most tightly bound candidate uremic toxin (KA) observed in the present study, it is not unreasonable to expect that dialytic removal of other uremic toxins might be achieved with the creation of a system that facilitates greater pH-mediated dissociation of protein-bound uremic solutes and increased dialyzer clearance.

In conclusion, we have demonstrated that a panel of uremic retention solutes exhibited a great range of binding affinity to presumed sites on albumin. The plasma concentrations of the three solutes identified as exhibiting the greatest binding affinity, KA, IS, and PCS, have been reported to be associated with increased incidence of cardiovascular disease in patients with chronic kidney disease, and therefore can be described as uremic toxins. The concentration of all of the uremic retention solutes declined during a single dialysis. This effect was predominantly attributable to solute removal. The concentration of free solute declined as a consequence of increased protein binding and dialyzer clearance. The finding that isohydric dialysis resulted in decreased binding of kynurenic acid and increased dialyzer clearance of the uremic toxin may be seen as a proof of principle and lead to efforts to further reduce pH of plasma passing through the dialysis cartridge to enhance dialyzer efficiency in clearing uremic toxins.

## Supporting information

S1 File An excel workbook with all original data for10 uremic solutes, pH and bicarbonate. (XLSX)
