# Correction: The effect of isohydric hemodialysis on the binding and removal of uremic retention solutes

**DOI:** 10.1371/journal.pone.0200980

**Published:** 2018-07-16

**Authors:** Aleksey Etinger, Sumit R. Kumar, William Ackley, Leland Soiefer, Jonathan Chun, Prabjhot Singh, Eric Grossman, Albert Matalon, Robert S. Holzman, Bjorn Meijers, Jerome Lowenstein

The second author’s name appears incorrectly. The correct name is: Sumit R. Kumar. The correct citation is: Etinger A, Kumar SR, Ackley W, Soiefer L, Chun J, Singh P, et al. (2018) The effect of isohydric hemodialysis on the binding and removal of uremic retention solutes. PLoS ONE 13(2): e0192770. https://doi.org/10.1371/journal.pone.0192770.
